# Reciprocal cross-species induction of outer membrane vesicle biogenesis via secreted factors

**DOI:** 10.1038/s41598-018-28042-4

**Published:** 2018-06-29

**Authors:** Alexander M. Horspool, Jeffrey W. Schertzer

**Affiliations:** 1 Department of Biological Sciences, Binghamton University, Binghamton, New York USA; 2 Binghamton Biofilm Research Center, Binghamton University, Binghamton, New York USA

## Abstract

Delivery of cargo to target cells is fundamental to bacterial competitiveness. One important but poorly understood system, ubiquitous among Gram-negative organisms, involves packaging cargo into outer membrane vesicles (OMVs). These biological nanoparticles are involved in processes ranging from toxin delivery to cell-cell communication. Despite this, we know comparatively little about how OMVs are formed. Building upon the discovery that the *Pseudomonas* Quinolone Signal (PQS) stimulates OMV biogenesis in *Pseudomonas aeruginosa*, we proposed a model where PQS interacts with the outer membrane to induce curvature and ultimately OMV formation. Though this model is well supported in *P. aeruginosa*, it remained unclear whether other organisms produce similar compounds. Here we describe the development of a tightly controlled experimental system to test the interaction of bacterially-produced factors with target cells. Using this system, we show that multiple species respond to PQS by increasing OMV formation, that PQS accumulates in the induced vesicles, and that other bacteria secrete OMV-promoting factors. Analysis of induced vesicles indicates that recipient-mediated mechanisms exist to control vesicle size and that relatedness to the producer organism can dictate susceptibility to OMV-inducing compounds. This work provides evidence that small molecule induced OMV biogenesis is a widely conserved process and that cross-talk between systems may influence OMV production in neighboring bacteria.

## Introduction

Outer Membrane Vesicles are small unilamellar structures released from Gram-negative bacteria that contain cargo such as virulence factors, enzymes, DNA, and communication signals^1–4^. OMV membrane composition is similar to the outer membrane of the producing organism, with an outer leaflet rich in lipopolysaccharide (LPS) and an inner leaflet composed primarily of phospholipid^5–7^. Across species, OMVs have been implicated in several behaviors that are important for pathogen resilience, competition and virulence. These include threat avoidance^6,8^, immune system interference^6^, toxin packaging and delivery^2,9,10^, antibiotic resistance via horizontal gene transfer^3,11^, and small molecule communication^4^.

Though much is known about the function of OMVs, the mechanism by which they are produced remains unclear. Historically, OMV biogenesis has been studied in several species by introducing membrane-perturbing agents (e.g. antibiotics, chelators, hydrophobic compounds) to bacterial cultures^12–16^. Consistent with this, several models of OMV biogenesis have been proposed to involve misregulation of cell wall or outer membrane remodeling^17–20^ that activates various membrane stress regulatory cascades^20,21^. Though past work with heterologous membrane-active compounds and treatments were shown to stimulate OMV formation, recent focus has shifted to include self-produced small molecules. The quorum-sensing molecule PQS (*Pseudomonas* Quinolone Signal) produced by *Pseudomonas aeruginosa* was discovered to be transported within *P. aeruginosa* OMVs^4^. Further investigation into the function of PQS indicated that it promotes the formation of OMVs in *P. aeruginosa* and can induce membrane vesicle formation without a requirement for either its receptor or *de novo* protein synthesis^4^. Biophysical examination of the interactions between PQS and various lipids gave evidence to suggest that PQS preferentially interacts with LPS in the outer membrane^22^. Building upon this information, we proposed that intercalation of PQS into the outer leaflet of the *P. aeruginosa* outer membrane causes asymmetric leaflet expansion leading to membrane curvature and ultimately OMV biogenesis^23^. We showed that PQS was capable of inducing curvature in surrogate erythrocyte membranes^23^, and recently demonstrated that the molecule must be transported to the outer membrane in order for OMV induction to take place in *P. aeruginosa*^24^. With a deeper understanding of the biophysical contributions to OMV biogenesis in *P. aeruginosa*, we were motivated to investigate whether small molecule-induced (SMI) OMV biogenesis might be a more broadly conserved mechanism.

Anecdotal evidence from our own laboratory as well as observations from the literature^25,26^ suggest that PQS may be capable of inducing OMV biogenesis when administered at high concentration to select organisms grown in rich medium. A recent study suggested that other unknown compounds fractionated from *P. aeruginosa* supernatants may have similar capabilities when given at very high concentration^26^. At the same time, a different class of self-produced hydrophobic signaling molecule (related to the diffusible signal factor of *Xanthomonas campestris*) has been associated with both increased (in *Stenotrophomonas maltophilia*^27^) and decreased (in *Xylella fastidiosa*^28^) production of OMVs, though in both of these cases the authors described their findings in the context of quorum signaling rather than physical interaction with the membrane. Observations such as these highlight the potential for SMI OMV biogenesis to be active across species and even to be involved in cross-species interactions.

With this work we set out to develop a rigorous and controlled system to test the cross-species effects of a known OMV-inducing compound and to lay the groundwork for identification of similar factors produced by other species. Using this system, we show that PQS is capable of OMV induction in other gammaproteobacteria under strict conditions, but that its effect is limited in more distantly related species. Properties of the induced OMVs indicate that PQS stimulates the formation of native-like vesicles in recipient strains, suggesting that features such as OMV diameter are controlled by the recipient organism rather than dictated by intrinsic properties of PQS. Finally, we identify cross-species (targeting *P. aeruginosa*) OMV-inducing activities in the supernatants of *Escherichia coli* and *Klebsiella pneumoniae*, supporting the hypothesis that SMI OMV biogenesis is active in multiple species.

## Results

### PA14 ΔpqsA mutant produces OMVs in response to PQS

**Figure 1:**
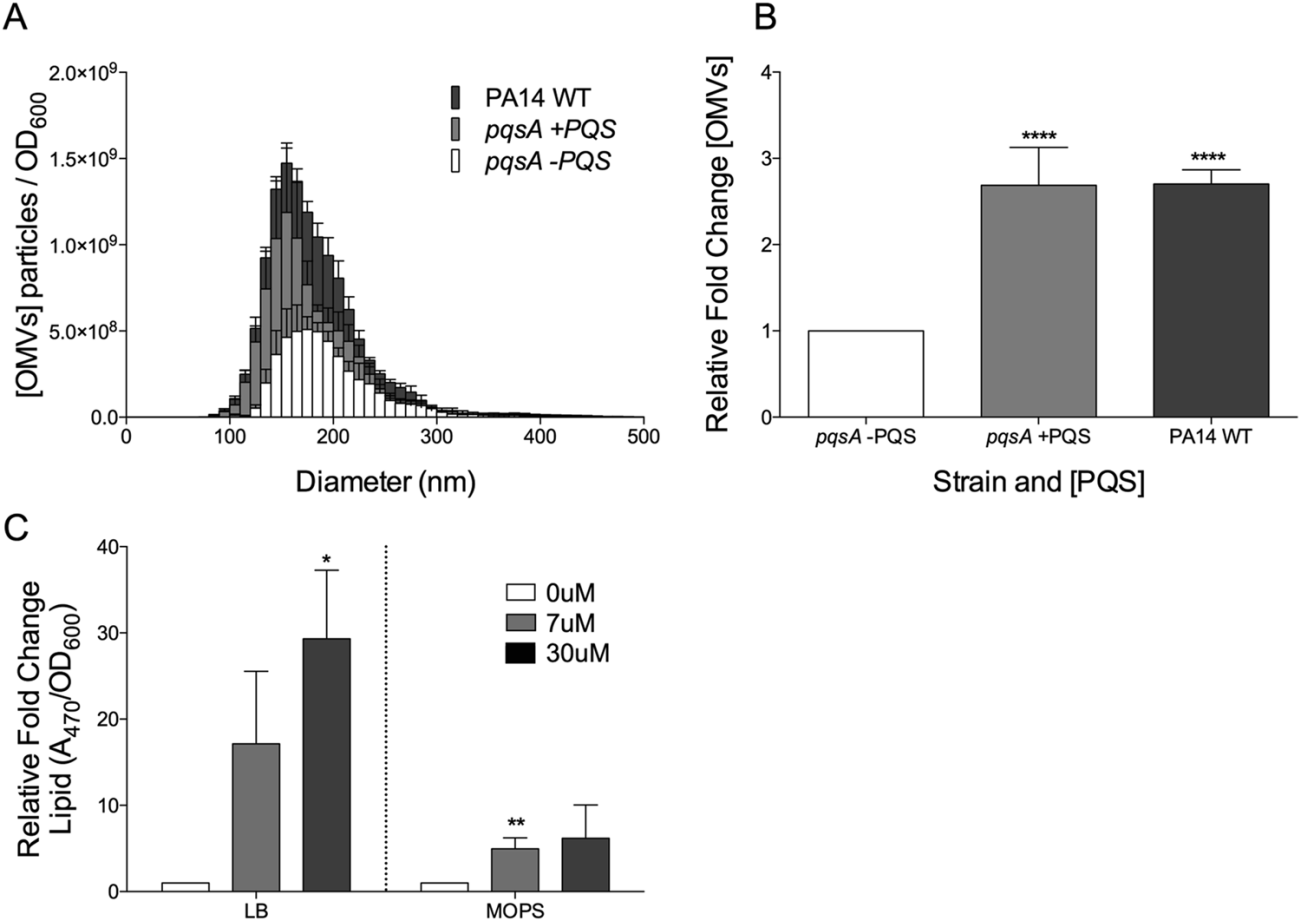
*P. aeruginosa ΔpqsA* was grown to early stationary phase in defined medium. Cells were then removed and exposed to exogenous PQS for 2 hours in fresh defined medium. OMVs were harvested by filtration (0.45 μm) followed by ultracentrifugation, then analyzed by NTA and lipid analysis. (**A**) Average concentration and size distribution of OMVs in *ΔpqsA* cultures +/− exogenously added PQS. (**B**) Fold increase in OMV production for *ΔpqsA* in the presence of exogenous PQS (relative to 0 µM PQS). In both (**A**) and (**B**), wild type PA14 OMV production under “−PQS” experimental conditions is included for reference. Exogenous [PQS] in “+PQS” = 6.7 ± 1.4 µM. (**C**) Analysis of PQS induced vesicles produced in rich (LB) or defined medium (MOPS) by lipid assay. Statistical significance was analyzed by one-tailed Student t-Test. (****p < 0.0001, **p < 0.01, *p < 0.05, n = 4).

In an effort to design a comprehensive and rigorous system to test the ability of PQS to induce OMV formation, we exposed a *P. aeruginosa* mutant defective in PQS production (*ΔpqsA*)^29,30^ to ~7 µM (see Methods) exogenous PQS for 2 hours during early stationary growth phase in limited defined medium. OMV production was then quantified by Nanoparticle Tracking Analysis (NTA). This setup gave unparalleled control over experimental conditions, but also presented a trade-off against overall OMV production. This is evidenced by the fact that wild type *P. aeruginosa* produced significantly fewer OMVs on its own (i.e. without exogenous PQS) when grown under our conditions than when grown in LB medium (Fig. S1). We selected the early stationary growth phase for administration of PQS as prior experiments have indicated that this is the stage at which *P. aeruginosa* maximally produces OMVs^24,31^. In line with previous literature, *P. aeruginosa ΔpqsA* exposed to exogenous PQS exhibited an increase in OMV production compared to *P. aeruginosa ΔpqsA* that received a mock exposure (2.7 ± 0.39 fold) (Fig. 1). This increase was nearly indistinguishable from the difference in OMV production between uninduced *ΔpqsA* and wild type PA14 (2.7 ± 0.16 fold) (Fig. 1B). Thus, exogenously added PQS given at low concentration for a short duration was capable of restoring OMV production to wild type levels in a mutant of the producer organism *P. aeruginosa*.

To corroborate the NTA analysis and provide information about the composition of the particles counted as OMVs, we also quantified OMV production using a biochemical approach developed for the quantification of membrane lipids^32^. Figure 1 shows that exogenous PQS addition produced a dose-dependent induction of OMVs in both rich and defined medium when measured by lipid analysis. As with natural OMV production (Fig. S1), the effect was greater in rich medium. It is noteworthy that the fold changes in response to PQS in defined medium are comparable between the two measurement techniques (2.7 ± 0.39 by NTA vs. 4.9 ± 1.29 by lipid assay). This suggests that the induced particles we measure by NTA are composed of membrane lipid and are in fact *bona fide* OMVs.

### Gammaproteobacteria produce OMVs in response to PQS

**Figure 2:**
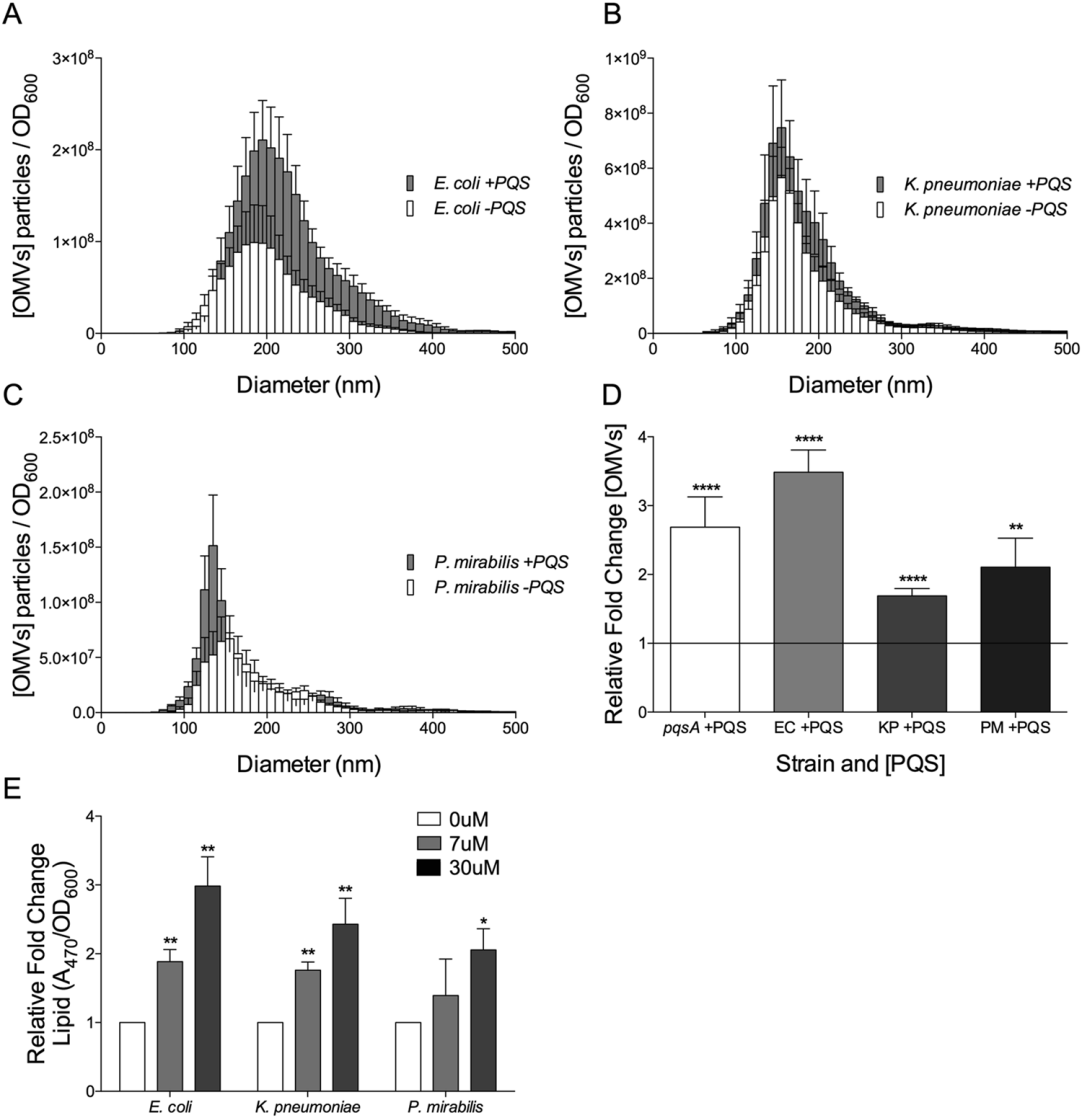
Cultures of (**A**) *E. coli*, (**B**) *K. pneumoniae*, and (**C**) *P. mirabilis* were grown to early stationary phase as described in Materials and Methods. Cells were then removed and exposed to exogenous PQS for 2 hours in fresh defined medium. OMVs were harvested by filtration (0.45 μm) followed by ultracentrifugation, then analyzed by NTA. (**D**) Fold increase in OMV production for all Gammaproteobacteria in the presence of exogenous PQS (relative to 0 µM PQS for each corresponding strain). (**E**) Relative fold increase of OMV production in response to PQS as measured by lipid assay. Exogenous [PQS] in “+PQS” = 6.7 ± 1.4 µM. EC = *E. coli*, KP = *K. pneumoniae*, PM = *P. mirabilis*. Statistical significance was analyzed by one-tailed Student t-Test. (****p < 0.0001, **p < 0.01, n = 4, *E. coli* n = 5).

To address the question of whether SMI OMV formation is a conserved process that is driven largely by biophysical interactions, we hypothesized that recipient cells of many species would respond to the known small molecule OMV inducer produced by *P. aeruginosa*. Several species of gammaproteobacteria (each of which has previously been reported to be capable of natural OMV production^21,33–35^) were tested for their response to exogenously added PQS by NTA and lipid analysis (Fig. 2). Cultures were grown to early stationary phase, washed, and exposed to PQS in fresh limited and defined exposure medium (or to a mock exposure medium lacking PQS). OMVs were then isolated and quantified as described in Materials and Methods. All species exhibited an increase in OMV production when exposed to PQS for 2 hours under these strict conditions (By NTA: *E. coli*, 3.5 ± 0.32 fold, *K. pneumoniae* 1.7 ± 0.11 fold, *P. mirabilis* 2.1 ± 0.42 fold) (Fig. 2). Lipid analysis revealed a dose-dependent response to PQS for each species and affirmed that the induced particles were composed of membrane lipids (Fig. 2E). This consistent increase in OMV production, observed across several species, suggests a widespread effect of PQS on OMV biogenesis. To ensure that the quantified vesicles were not the product of cell lysis/disintegration, we routinely performed an assay to detect the presence of contaminating inner membrane (i.e. succinate dehydrogenase activity) in culture supernatants. No succinate dehydrogenase activity was measured in the supernatant from any strain exposed to PQS (Fig. S2), which confirms that exogenous addition of PQS did not induce appreciable cell lysis/disintegration in recipient cultures under our assay conditions.

### Induced OMVs resemble natural OMVs in size

**Figure 3:**
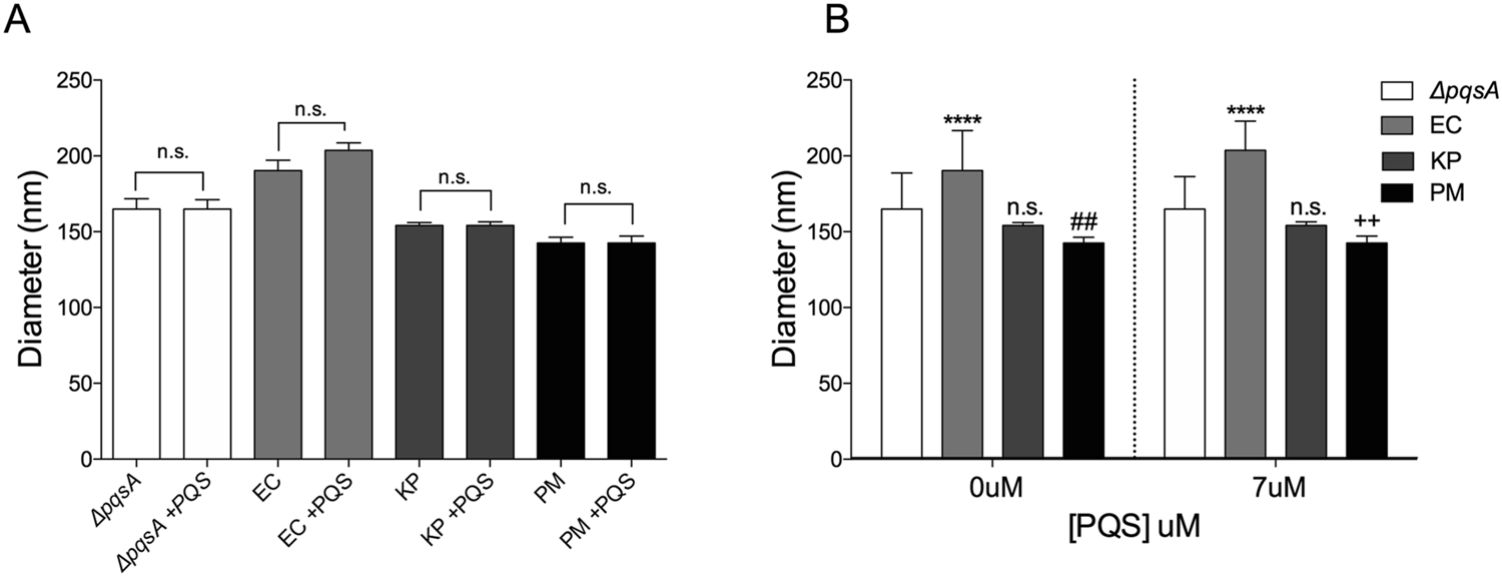
Comparison of the mode diameter of OMVs induced by PQS in each species. (**A**) Differences in OMV size +/− PQS *within* each species. No statistically significant difference was observed for any species. (**B**) Size comparisons organized to highlight differences *between* species both in the absence and presence of PQS. Asterisks represent statistically significant differences in mode OMV diameter compared to *ΔpqsA* under the same condition. Exogenous [PQS] in “+PQS” = 6.7 ± 1.4 µM. Size was measured by NTA and statistical significance was assessed by one-tailed Student t-test. (n.s. = p > 0.05, **p = 0.0079, ^##^p = 0.0047, ^++^p = 0.0039, ****p < 0.0001, n = 12, *E. coli* n = 15).

For each species of gammaproteobacteria tested, we analyzed the characteristics of the PQS-induced OMVs. Nanoparticle tracking analysis (NTA) allowed us to record the entire distribution of vesicle diameters produced in the presence and absence of exogenous PQS, and to identify the most commonly produced diameter under each condition (the mode). Figure 3 shows the mode vesicle diameter for PQS-induced OMVs from several different recipient species. Counter to our expectations, OMVs produced in response to PQS did not have a uniform *Pseudomonas*-like size across species. Rather, PQS-induced OMVs showed no difference in size compared to naturally-produced vesicles for any of the species tested (Fig. 3A). When compared across species, it was clear that each species naturally produced OMVs of a characteristic size and that PQS induced the production of OMVs of this characteristic size for each recipient species (Fig. 3B). This suggests that, although PQS is clearly capable of inducing OMVs in these recipient species, there appear to be endogenously-mediated mechanisms that contribute to OMV size determination. In other words, OMV size was not dictated solely based on the intrinsic properties of PQS.

### Induced OMVs from *P. aeruginosa* contain PQS

**Figure 4:**
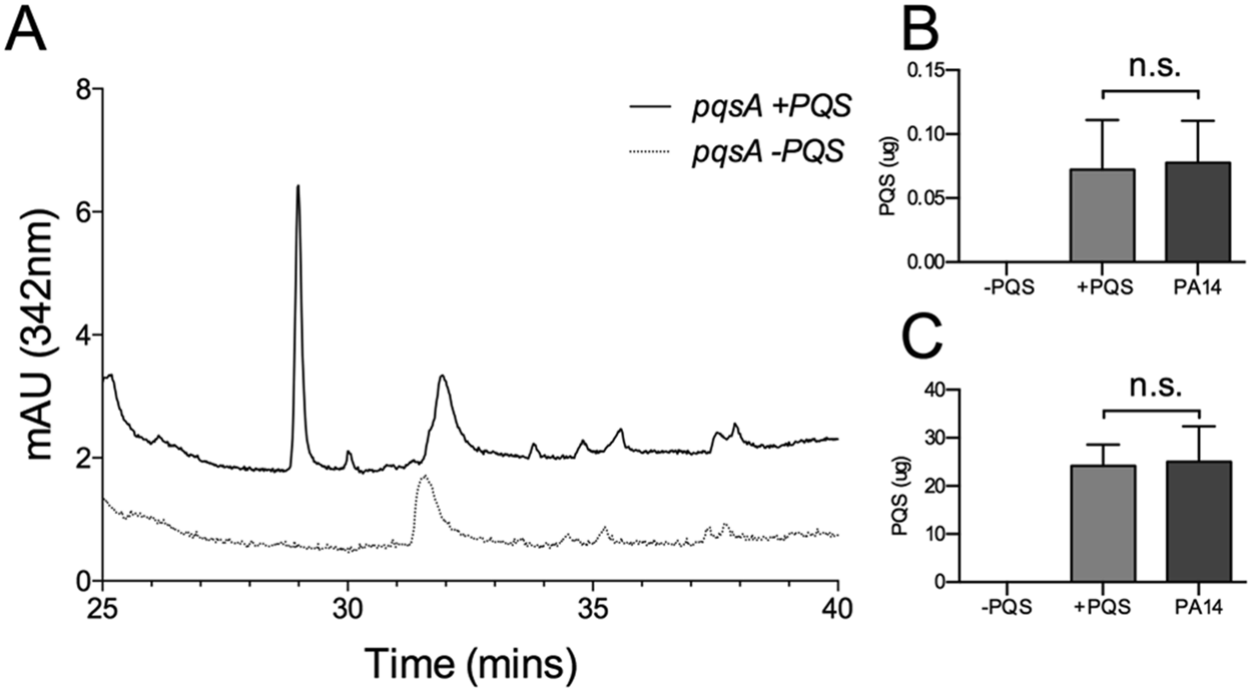
PQS was extracted from isolated cells or OMVs using a 1:1 ethyl acetate ratio. The organic layer was dried under Nitrogen and the resulting sample was resuspended in 50% methanol. PQS was analyzed using reverse-phase HPLC. (**A**) Chromatogram of compounds extracted from *ΔpqsA* (+/− PQS) OMVs. The quantity of PQS recovered from (**B**) OMVs and (**C**) cells was measured for *ΔpqsA* +/− PQS and wild type PA14 grown under the “−PQS” condition. PQS retention time: 28.9 minutes. Exogenous [PQS] in “+PQS” = 6.7 ± 1.4 µM. Statistical significance was analyzed by one-tailed Student t-Test. (n.s. p > 0.05, n = 3).

In order to induce OMV formation, the Bilayer-Couple model predicts that PQS must intercalate into the outer leaflet of the bacterial outer membrane, which is then shed as OMVs. If this is true, PQS should be recoverable from induced OMVs. To test this hypothesis, the *P. aeruginosa ΔpqsA* mutant was exposed to exogenous PQS or a mock treatment and PQS was extracted from the recovered cells and OMVs. The amount of PQS recovered from both cells and OMVs was determined by HPLC. Figure 4 shows that *P. aeruginosa ΔpqsA* OMVs and cells contained PQS after exposure to exogenous PQS. The amount of PQS recovered from both *P. aeruginosa ΔpqsA* OMVs and cells was similar to the amount of PQS recovered from wild type PA14 OMVs and cells after a mock exposure under identical experimental conditions (i.e. the natural distribution seen in wild type PQS-producing cells) (Fig. 4B,C). The results presented here demonstrate that packaging of PQS into *ΔpqsA* OMVs and cells was restored to wild type levels when PQS was delivered exogenously at low concentration.

### Induced OMVs from other Gammaproteobacteria contain PQS

**Figure 5:**
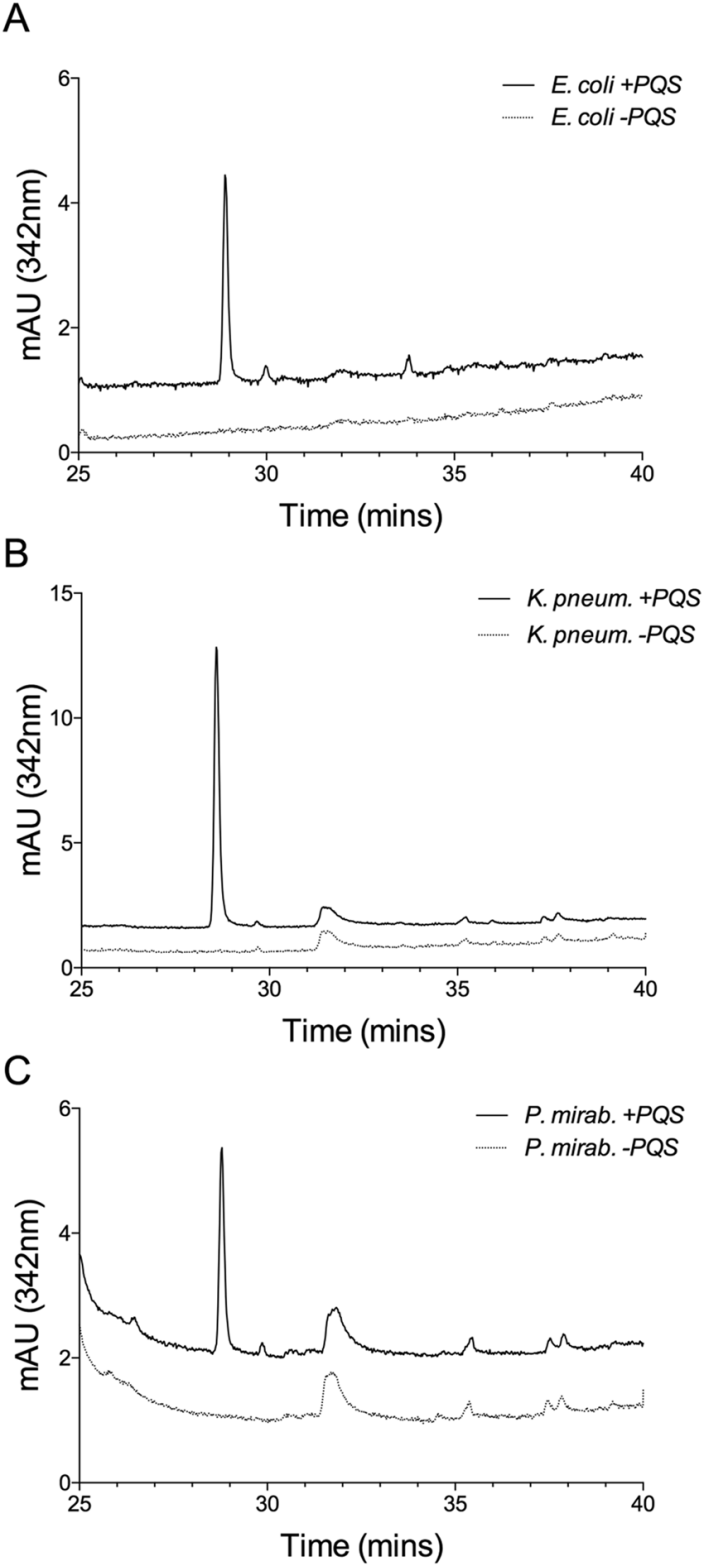
PQS was extracted from OMVs using a 1:1 ethyl acetate ratio. The organic layer was dried under Nitrogen and the resulting sample was resuspended in 50% methanol. PQS was analyzed using reverse-phase HPLC as in Fig. 4. Chromatograms show compounds extracted from OMVs of (**A**) *E. coli* (+/−PQS), (**B**) *K. pneumoniae* (+/−PQS), and (**C**) *P. mirabilis* (+/−PQS). PQS retention time: 28.9 minutes. Exogenous [PQS] in “+PQS” = 6.7 ± 1.4 µM.

Since we showed that PQS induces OMV production in several strains of gammaproteobacteria and that PQS can be recovered in induced *P. aeruginosa ΔpqsA* OMVs, we investigated whether PQS induced vesicles from other recipient gammaproteobacteria contained PQS. We hypothesized that if these OMVs were produced by the same mechanism, then PQS should be recoverable from the induced OMVs of each species, similar to how PQS was recoverable from *P. aeruginosa* OMVs. We tested each species for PQS induced vesicle production as previously described, extracted the resulting OMVs for PQS, and analyzed the extracts by HPLC. We were able to identify PQS in the induced OMVs from each strain tested (Fig. 5). This demonstrates that a small molecule inducer (namely PQS) is capable of interacting with the outer membranes of an array of gammaproteobacteria to induce its own packaging into OMVs.

### Alphaproteobacteria do not respond to PQS

**Figure 6:**
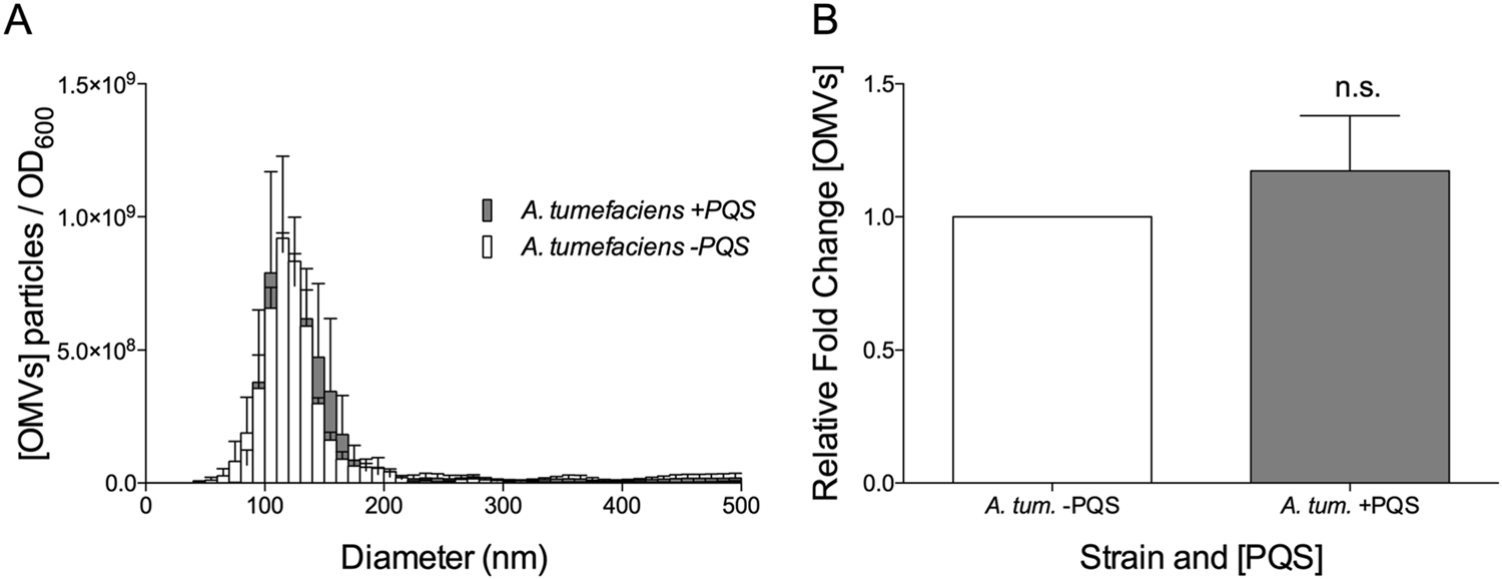
Cultures of *A. tumefaciens* were grown to early stationary phase in defined medium. Cells were removed and exposed to exogenous PQS for 2 hours in fresh defined medium. OMVs were harvested by filtration (0.45 μm) followed by ultracentrifugation, then analyzed by NTA. (**A**) Average concentration and size distribution of OMVs +/− exogenously added PQS. (**B**) Relative fold increase of OMV production (relative to 0 µM PQS). Exogenous [PQS] in “+PQS” = 6.7 ± 1.4 µM. Statistical significance was analyzed by one-tailed Student t-Test. (n.s. p > 0.05, n = 3).

Our results demonstrate that PQS induced OMV formation can be achieved under very restrictive conditions using several recipient species of bacteria. To examine how widespread this phenomenon is, we selected additional recipient species that are more distantly related to *P. aeruginosa*. We tested two species of alphaproteobacteria for PQS-induced OMV formation by growing each culture to early stationary phase and exposing them to PQS in our limited defined exposure medium, as we had done with the gammaproteobacteria above. Our results showed that administration of PQS to *Agrobacterium tumefaciens* did not affect the production of OMVs (Fig. 6) (p = 0.17). Treatment of *Caulobacter crescentus* with exogenous PQS yielded a similar non-response (not shown). These results suggest that the ability of an organism to respond to a given small molecule OMV inducer may be limited to more closely related species.

### Raw supernatants from other species induce OMVs in *P. aeruginosa*

**Figure 7:**
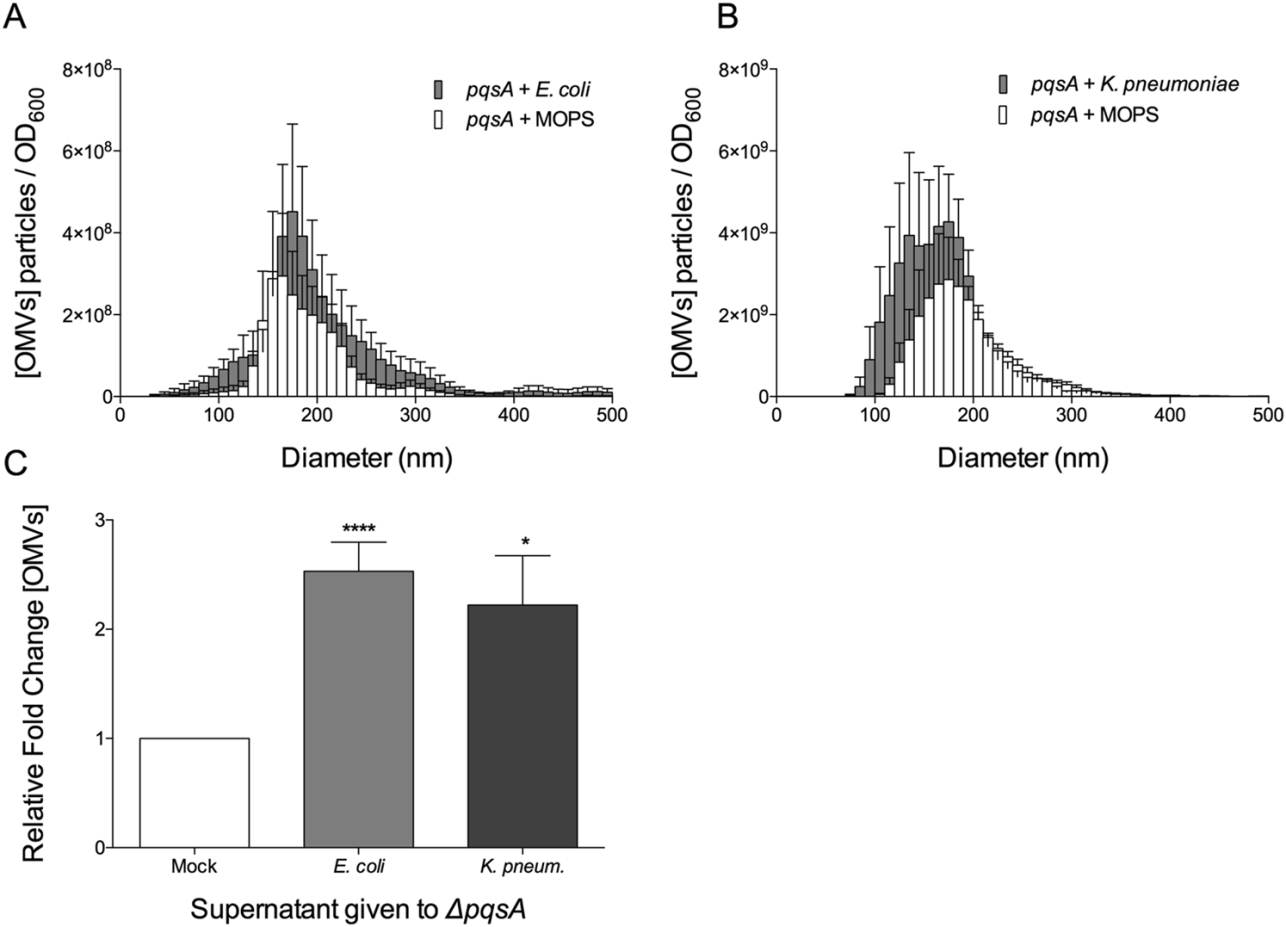
*P. aeruginosa ΔpqsA* was grown to early stationary phase in defined medium. Cells were removed, reconstituted in fresh exposure medium and treated with 2.2 × concentrated supernatant from (**A**) *E. coli* or (**B**) *K. pneumoniae*. (**C**) Comparison of relative fold increase of OMVs produced by the *ΔpqsA* mutant in response to concentrated supernatant (compared to mock induced cultures). Statistical significance was analyzed by one-tailed Student t-Test. (****p < 0.0001, *p = 0.0121, *E. coli* n = 4, *K. pneumoniae* n = 5).

Until now, PQS has been the only known self-produced factor capable of inducing OMV biogenesis. This perceived uniqueness has been a significant limitation in our ability to investigate whether small molecule induced OMV biogenesis is a generally conserved mechanism. To begin to address this, we set out to look for OMV-inducing activity in the culture supernatants from other bacteria. Supernatants from cultures of *E. coli* and *K. pneumoniae* were harvested at early stationary phase and concentrated 2.2× by lyophilization. Concentrated supernatant from either *E. coli* or *K. pneumoniae* was administered to early stationary phase *P. aeruginosa* Δ*pqsA* in fresh exposure medium (as described in Materials and Methods). Our results show that Δ*pqsA* produced 2 to 3-fold more OMVs in response to concentrated supernatants of *E. coli* (2.5 ± 0.27 fold) and *K. pneumoniae* (2.2 ± 0.45 fold) (Fig. 7). Under our experimental conditions, this response was comparable to the response of *ΔpqsA* to its own endogenously-produced OMV inducer, PQS. This provides exciting new evidence to suggest that there are OMV promoting factors produced by other bacteria and that secretion of such factors to induce OMV biogenesis may be a mechanism that extends beyond only *P. aeruginosa*.

## Discussion

OMV biogenesis has been described as a dedicated secretion system and is ubiquitous among Gram-negative organisms. We developed the Bilayer-Couple Model^23^ to describe OMV biogenesis in *P. aeruginosa* that is induced by interactions between a self-produced small molecule (PQS in this case) and the producer cell’s outer membrane. The model has been well supported by genetic studies^4^, culture ‘add-back’ experiments^4,23,25^, theoretical modeling^36^ and studies with surrogate membranes^23^. The fact that *P. aeruginosa* is unique in synthesizing PQS, however, has meant that SMI OMV biogenesis has been difficult to generalize to other organisms. With this work, we show that short term exposure to PQS at low concentration under controlled conditions induces OMV biogenesis in multiple Gram-negative species. Analysis of the vesicles suggests that PQS stimulated production of ‘native’ vesicles in the recipient organisms, rather than causing them to produce *Pseudomonas*-like vesicles. OMV-inducing activities were identified in the supernatants of the recipient species as well. This reciprocal cross-stimulation supports the premise that small molecule induced OMV biogenesis is a broadly conserved mechanism.

To systematically study this phenomenon, we needed to first develop a rigorous yet flexible experimental system that controlled for some known difficulties in working with PQS (and other membrane-intercalating molecules). PQS is hydrophobic^23,37,38^ and is prone to aggregate in aqueous solution, with maximum solubility reported in the single digit micromolar range^37,38^. Previous work in this area has relied largely on delivering PQS at relatively high concentration in a carrier solvent^4,23,25,39^. This practice has proven invaluable in the past, but runs the risk of creating aggregates that could be misidentified as OMVs. Our new approach was to pre-equilibrate PQS into aqueous solution, discard insoluble PQS, and then add cells to this medium. Routine HPLC quantification of the PQS concentration in our pre-equilibrated medium yielded a value of 6.7 ± 1.4 μM, in good agreement with reported limits for PQS solubility^37,38^ and on the low end of concentrations reported for whole-culture production in rich medium^24,37^. To further reduce the risk of misidentification of PQS aggregates as OMVs, we used defined exposure medium absent iron and other divalent cations in place of undefined rich medium. Thus, aggregate nucleation via PQS chelating activity^39,40^ was minimized. Pre-equilibrated exposure medium was routinely evaluated using NTA to ensure the absence of particles before use in experiments. Similar analysis was also used to quantify OMV production after exposure to PQS or spent culture supernatant. NTA was an ideal choice for this analysis because it is unbiased toward the chemical composition of the outer membrane surface (superior to common LPS quantification methods^41^) and measures the physical presence of OMV particles rather than relying on hydrophobic membrane lipids facilitating movement of a dye into the organic phase during extraction (superior to the common lipid assay^32^). NTA also gives valuable size distribution information in addition to the number of OMV particles. By carefully considering all of these factors, and limiting the exposure of cells to PQS both in time and concentration, we have developed a strict yet flexible system focused as much as possible on isolating the biophysical contributions to small molecule induced OMV biogenesis.

Using our novel system, we were able to show that PQS induces OMV production in *P. aeruginosa*, *E. coli*, *K. pneumoniae*, and *P. mirabilis*. Beginning with *P. aeruginosa*, we confirmed that exogenous addition of PQS can complement the loss of OMV production in a PQS-defective mutant back to wild type levels. This is significant because, to our knowledge, this has never been shown using such a controlled system and after exposure to such low concentrations for so little time. Despite this intentional strictness, the magnitude of the fold inductions measured here for exogenous PQS agree well with recent reports examining other contributors to OMV biogenesis^18,28,29,42^. The fact that PQS was able to induce OMV formation in at least three Gram-negative organisms that do not produce PQS themselves gives strong support to the hypothesis that this response is governed by physical interactions with the membrane and is not due to receptor-mediated responses. In a similar vein, careful consideration was taken to ensure that the observed PQS responses were not simply the result of cell lysis in our system (as has been proposed as a mechanism for extracellular vesicle production in specialized biofilm models^43^).

The Bilayer-Couple Model states that small molecule OMV inducers interact with target membranes to induce membrane curvature and initiate OMV biogenesis. A reasonable prediction, then, is that the inducing agent should be packaged during this process and therefore be recoverable from harvested OMVs (as has been reported for PQS in *P. aeruginosa*^4^). We harvested both cells and OMVs from treated cultures to assess whether exogenous PQS had interacted at all with the recipient cells and whether it had been packaged within the induced OMVs. For each recipient species, PQS was recovered from the induced OMVs. In addition, control experiments showed that exogenous addition of PQS to the *P. aeruginosa ΔpqsA* mutant yielded a distribution of PQS between cells and OMVs that was indistinguishable from wild type. It is interesting to note that the proportion of PQS found in OMVs was much lower than has been reported elsewhere^4^. The strictness of our system (in lower PQS concentration, shorter exposure time, limited exposure medium, and lack of a carrier solvent) likely contributed to this difference. This point is supported by the fact that the wild type also behaved very differently under our conditions and that others have also seen substantial drops in PQS packaging when comparing endogenous production to exogenous addition^4,24^. It is interesting to note that the proportion of exogenously added PQS packaged into OMVs in our system was similar to the proportion of total OM previously reported to be released as OMVs in planktonic cultures (0.75–2.5%)^33^. This suggests that when PQS is added exogenously it may intercalate evenly throughout the OM and subsequently become packaged into OMVs in close proportion to the amount of OM released. Naturally-produced PQS, which is exported through a still-unknown mechanism, is reported to become much more concentrated into OMVs^4,24,25^. Our own experiments agree with this finding^24^. Taken together, these results are consistent with the prediction that a small molecule inducer like PQS must intercalate into a target membrane to initiate OMV biogenesis, which results in its subsequent packaging into the resulting vesicles. Our analysis also highlights the importance of native export for optimal OMV production, and may hint at the requirement for additional endogenous systems to replenish lost OM or facilitate continued vesiculation in other ways. We are currently investigating the kinetics of SMI OMV biogenesis over shorter timescales and under less restrictive conditions. We expect these studies will give insight into these questions.

Although all the gammaproteobacteria tested here responded to PQS by increasing OMV production, there were intriguing differences noted when the vesicles were examined in greater detail. Unexpectedly, we found that PQS-induced OMVs in non-*Pseudomonas* species more closely resembled the recipient species’ naturally produced vesicles than those of *P. aeruginosa*. This finding demonstrates that the physical properties of the small molecule inducer (i.e. preferred membrane curvature) are not the sole governing factors that direct OMV biogenesis. Rather, species-specific processes must exist to control at least some characteristics, such as the size distribution of released vesicles. The level of response to PQS in terms of increased OMV production also differed between species, with *P. aeruginosa* and *E. coli* being stronger responders than *K. pneumoniae* and *P. mirabilis*. Aside from *P. aeruginosa*, where a PQS-deficient mutant was used, it is important to note that native OMV production was not hindered in any way for the other species. This meant that, depending on species, there might only have been a small window between ‘normal’ OMV production (0 μM PQS) and the maximum possible PQS-induced OMV production. Because OMV biogenesis may be a saturable process in other organisms, as we saw it was for wild type *P. aeruginosa*^24^, we expected to see a response to PQS in the recipient species that was weaker than the response of the *ΔpqsA* mutant. Instead, we observed both stronger and weaker responses. The fact that *E. coli* was the strongest responder suggests that something about this species makes it particularly sensitive to SMI OMV biogenesis. On the other hand, the remaining species showed varying degrees of responsiveness to PQS. We are very interested in the basis for these differences and suspect that variations in surface chemistry affect PQS association with LPS and subsequent ability to induce OMV biogenesis. In addition to opening a new and interesting avenue for study, the fact that multiple organisms respond differently to PQS solidifies the idea that PQS has a targeted effect on membranes and does not simply act as a non-specific detergent.

To gather more information on the effects of surface structure on SMI OMV biogenesis, we asked whether organisms more distantly related to *P. aeruginosa* would respond to PQS by increasing OMV production. Although members of the alphaproteobacteria class are known to produce OMVs^3,44,45^, two species tested here (*A. tumefaciens*, *C. crescentus*) showed no enhancement of OMV production in response to PQS. As mentioned above, it is possible that these organisms were already operating at maximum production before PQS addition and therefore were incapable of responding. Until more is known about OMV biogenesis in these species (allowing natural production to be reduced), it will be impossible to rule out this possibility. A more interesting and likely explanation, we believe, is that known differences in envelope composition and structure^46^ between this class and the responders described above rendered their outer membranes insensitive to PQS-induced vesiculation. This is an area of major interest for us and we have seen, even using only *P. aeruginosa*, that growth under different conditions can affect OMV production in interesting ways^24^. Recent reports also appear to support the hypothesis that the chemical structure of membrane lipids can affect OMV formation^42,47^.

A goal of this work was to investigate the possibility that SMI OMV biogenesis was operative in species other than *P. aeruginosa*. Our results make it clear that several other species are capable of responding to PQS by producing OMVs, and that this receptiveness appears to correlate with their relatedness to *P. aeruginosa*. The diversity of response to PQS and its limitation to more closely related organisms lend biological relevance to what can now be viewed as a more widespread phenomenon. Perhaps the most exciting finding from our study was that these same recipient organisms secrete factors into the supernatant that can cross-stimulate the *ΔpqsA* mutant to increase OMV production. While reports exist of non-PQS small molecules inducing OMV production in *Pseudomonas* species^15,16^, to our knowledge this marks the first report of such activities that are naturally produced and secreted by other organisms. As was successfully done for quorum sensing molecules in the past^48–50^, we are now set to purify and characterize these activities. The fact that supernatants concentrated only ~2-fold elicited strong cross-species OMV responses gives us confidence that this can be achieved using our system as a guide.

Despite its importance, OMV biogenesis remains a poorly understood phenomenon. With this work, we have demonstrated that SMI OMV biogenesis, as typified by the *P. aeruginosa* PQS system, is possible and likely occurs in at least a subset of other Gram-negative organisms. The ecological and medical implications of reciprocal cross-species induction of OMVs between species, especially among ESKAPE pathogens as demonstrated here, remains to be studied in detail and is of major interest to our group. With the concepts and tools developed in this work, we are poised to answer these and many other outstanding questions about the nature of OMV biogenesis across species.

## Materials and Methods

### Strains and Reagents

The gammaproteobacteria used in this study were *P. aeruginosa* PA14^51^ & *ΔpqsA* (a kind gift from Dr. Marvin Whiteley)*, E. coli* ATCC 11775*, K. pneumoniae* ATCC 10273 and *P. mirabilis* ATCC 25933 (kind gifts from Dr. Claudia Marques). The alphaproteobacteria used in this study were *A. tumefaciens* C58 and *C. crescentus* CB15 (kind gifts from Dr. Clay Fuqua). Each strain was inoculated at OD_600_ 0.01 in MOPS-Succinate (50 mM MOPS, 93 mM NH_4_Cl, 43 mM NaCl, 2 mM KH_2_PO4, 3.6 mM FeSO_4_, 1 mM MgSO_4_, 30 mM Succinate, pH 7.2) (Δ*pqsA* and *E.coli*), MOPS-Glucose (50 mM MOPS, 93 mM NH_4_Cl, 43 mM NaCl, 2 mM KH_2_PO4, 3.6 mM FeSO_4_, 1 mM MgSO_4_, 30 mM Glucose, pH 7.2) (*K. pneumoniae*), LB Miller broth (*P. mirabilis*), *A. tumefaciens* minimal medium^52^ (*A. tumefaciens*) or M2 minimal salts medium^53^ (*C. crescentus*) and grown at 37 °C (gammaproteobacteria) or 28 °C (alphaproteobacteria). PQS was purchased from Sigma-Aldrich.

### Preparation of PQS-containing medium

For this work, great care was taken to expose cells to PQS under condition where the risk of PQS aggregation was minimized. To achieve this, washed cells were added to pre-equilibrated PQS-containing medium rather than adding PQS to medium containing cells. PQS in methanol carrier solvent (Optima grade, Fisher) was added to base exposure medium (50 mM MOPS, 93 mM NH_4_Cl, 43 mM NaCl, 2 mM KH_2_PO4, pH 7.2) to a final concentration of 40 μM. This medium was allowed to incubate at 37 °C for 8–12 hr before use. PQS aggregates were separated from the solution and the resulting ‘PQS-solubilized’ medium was used for cell exposure experiments. Analysis by HPLC revealed that the ‘PQS-solubilized’ medium generated by this procedure contained 6.7 ± 1.4 μM PQS. Routine analysis by NTA confirmed that ‘PQS-solubilized’ medium was devoid of PQS aggregates.

### Culture growth conditions and PQS exposure

Cultures were inoculated into 25 mL fresh defined growth medium from overnight cultures at an OD_600_ of 0.01 and grown to early stationary phase. The cells were pelleted at 5000 g for 15 minutes at 4 °C using a Sorvall Legend XTR centrifuge F15 rotor. Cells were then resuspended in 25 mL of fresh ‘PQS-solubilized’ medium (see above) or mock PQS medium to which only the methanol carrier solvent had been added. Cultures were exposed for 2 hours at 37 °C (or 28 °C for alphaproteobacteria) before cells and OMVs were harvested for analysis.

### Isolation of outer membrane vesicles

Cultures were centrifuged at 15,000 g for 15 minutes at 4 °C. The supernatant was collected and filtered through a 0.45 μm filter. To isolate OMVs, 20 mL of supernatant was ultracentrifugated (Thermo Scientific S50-A rotor) at 209,438 g for 1.5 hours at 4 °C. Pelleted vesicles were resuspended in 500uL of PBS (137 mM NaCl, 10 mM Na_2_HPO_4_, 2.7 mM KCl, 2 mM KH_2_PO_4_) and kept at 4 °C until analyzed.

### PQS purification and quantification

PQS was extracted from media, cells, or OMV preparations via 1:1 addition of acidified ethyl acetate (0.1 mL/L acetic acid). The organic phase was removed and dried under nitrogen gas. Dried samples were resuspended in 50% methanol (Optima grade, Fisher). PQS was quantified using HPLC by reverse-phase chromatography on a Poroshell 120 EC-C8 4.6 mm × 150 mm 2.7 μm column, using a stepped linear gradient of 40% methanol in water to 100% methanol, acidified with 5.2 mM citric acid (pH 2.5), at a flow rate of 0.5 ml/min. Absorbance data was collected by photodiode array and the traces corresponding to λ_max_ for PQS (342 nm) are depicted. Total mass of PQS was calculated by comparison to known standards.

### Concentration and administration of supernatants

Bacteria were inoculated in 300 mL of defined growth medium at an OD_600_ of 0.01 and grown to early stationary phase. The cultures were centrifuged at 15,000 g for 15 minutes at 4 °C and the supernatants were harvested and filtered through a 0.45 μm filter. Filtered supernatants were frozen at −80 °C and lyophilized using a Labconco FreeZone 2.5 Plus Lyophilizer. Lyophilized samples were reconstituted in 10 mL of MOPS-Succinate and stored at 4 °C. 25 mL *ΔpqsA* cultures were inoculated at OD_600_ 0.01 in defined medium, grown to early stationary phase, and were induced by addition of 2 mL of the concentrated supernatants.

### Nanoparticle Tracking Analysis (NTA)

OMVs were quantified by direct nanoparticle counting. Pelleted vesicles were resuspended in 500uL PBS and diluted to obtain 15 to 100 particles per frame (as per manufacturer’s instructions). Vesicle size and concentration were analyzed using a NanoSight NS300 system and its corresponding Nanoparticle Tracking and Analysis software (NTA 3.1). Optimal settings for camera level, gain and detection threshold (12, 1 and 25, respectively) were manually programed and were held constant for all analyses. Each sample was analyzed three times for 30 seconds at 25 °C. Frame sequences were analyzed under consistent manual particle detection and tracking parameters. Values were normalized to the OD_600_ of the extracted culture.

### OMV Quantification Analysis

Particle concentration for each NTA run was normalized to culture OD_600_ and histograms were constructed based upon hydrodynamic diameter as calculated by NTA software. To calculate overall relative fold increase (RFI), all histogram bins referring to particles 50–300 nm (biologically relevant size range) that also contained >1,000,000 particles (instrument detection threshold) were first compared pairwise + vs. − PQS. The average of the RFIs for each histogram bin was then taken across the included portion of the distribution to generate an overall RFI for that experiment. This method was chosen to capture aspects of changes to both the overall particle number and the shape of the distribution. Comparable results were obtained whether the RFI was calculated using the total number of particles (of all sizes) or only the bin containing the mode particle size (see Fig. S3).

### Lipid Assay

OMVs were quantified by lipid content according to previously published protocols^54,55^. Following ultracentrifugation, OMV pellets were resuspended in MV buffer. Vesicles were extracted 1:1 with chloroform. The organic layer was removed and combined 1:1 with ammonium ferrothiocyanate solution (23.03 g/L FeCl_3_∙6H_2_O, 30.4 g/L NH_4_SCN), and mixed thoroughly. The organic layer was removed and analyzed for absorbance at 470 nm. Absorbance values were normalized to the OD_600_ of the extracted culture.

### Succinate Dehydrogenase Assay

Supernatants were separated from cells by centrifugation (15,000 × g for 15 min). Control samples were lysed via sonication (5.25 minutes at 30% amplitude by a Branson Digital Sonifier). SDH activity in culture supernatants and in control samples were routinely measured and compared to ensure that cell lysis/disintegration was not appreciable at the time of OMV harvest. The SDH assay was a modification of the method of Kasahara and Anraku^56^. Reactions were carried out in a 96-well plate (Falcon) in a total volume of 200 uL and were comprised of 50 mM Tris-HCl (pH 8.0), 4 mM potassium cyanide (KCN), 0.04 mM 2,6-dichlorophenolindophenol (DCPIP), 0.2 mM phenazine methosulfate (PMS), 40 mM disodium succinate, and 5 μl of sample. The reaction mixture without sample, DCPIP, or PMS was incubated for 5 min at room temperature. Following this incubation period, the supernatant (or sonicated control) sample was added to the mixture and allowed to acclimate for 5 min. Lastly, DCPIP and PMS were added in this order to initiate the reaction. SDH activity was quantified by measuring the decrease in absorbance at 600 nm over time at 25 °C using a Tecan Infinite M-200 Pro.

### Availability of Materials and Data

Materials and data generated in this study are available from the corresponding author upon reasonable request.

## Electronic supplementary material

Supplementary Info
