# Supplementary material for: Reciprocal cross-species induction of outer membrane vesicle biogenesis via secreted factors
Source: Sci Rep. 2018 Jun 29;8:9873. doi: 10.1038/s41598-018-28042-4 (PMC6026191; doi:10.1038/s41598-018-28042-4)
Supplement: Supplementary file 1 — Supplementary Info [file 41598_2018_28042_MOESM1_ESM.docx]

**Reciprocal cross-species induction of outer membrane vesicle biogenesis via secreted factors.**

Alexander M. Horspool^1,2^ and Jeffrey W. Schertzer^1,2,*^

^1^Department of Biological Sciences, Binghamton University, Binghamton, New York, USA

^2^Binghamton Biofilm Research Center, Binghamton University, Binghamton, New York, USA

*Correspondence should be addressed to J.W.S. ([jschertz@binghamton.edu](mailto:jschertz@binghamton.edu))


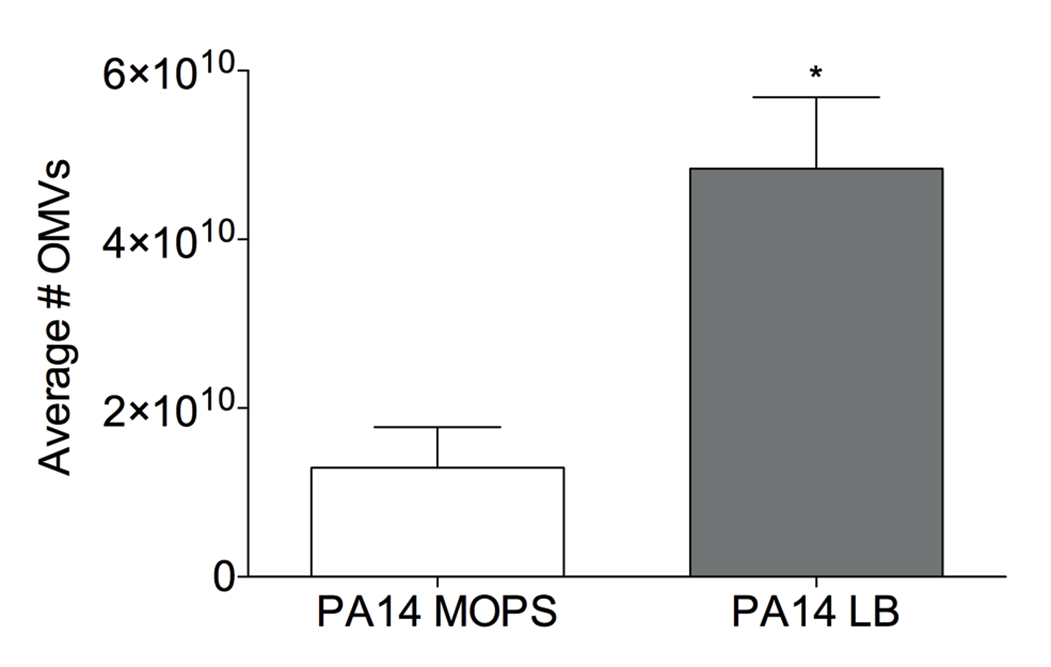


**Figure S1:** Wild type PA14 grown in MOPS-Succinate defined medium produces fewer OMVs than PA14 grown in LB. Cultures were grown to late exponential phase and OMVs were harvested by ultracentrifugation as described in Material and Methods. Analysis was performed by NTA and statistical significance was measured by a one-tailed Student t-test (* p = 0.0138).


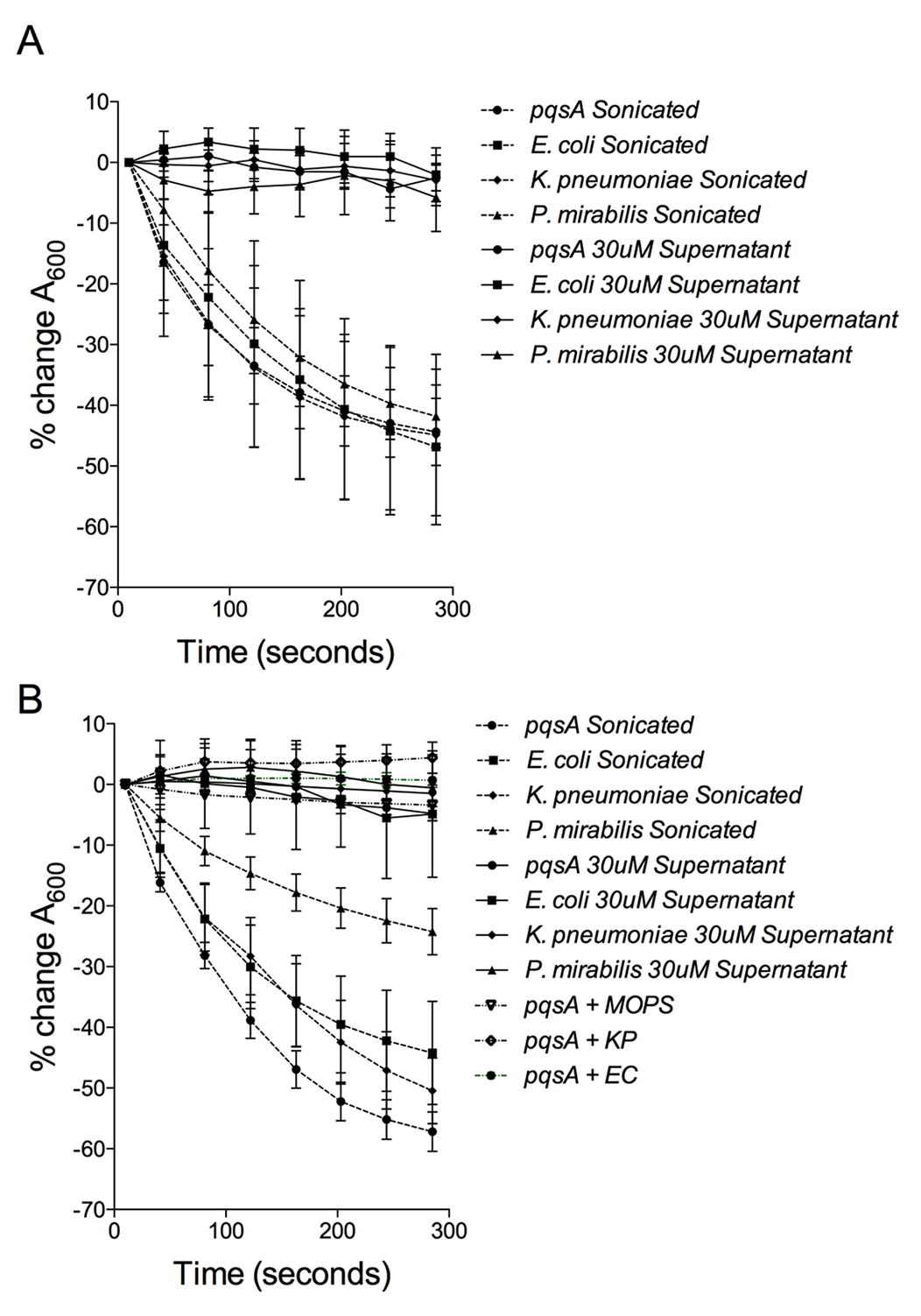


**Figure S2:** Culture supernatant was tested for lysis by assessing succinate dehydrogenase activity as measured by % decrease in A_600_ over time for each strain or in response to exogenous PQS or bacterial supernatant in (a) rich medium (LB) or (b) defined medium (MOPS). Cultures disrupted by sonication served as positive controls for each recipient species.

**
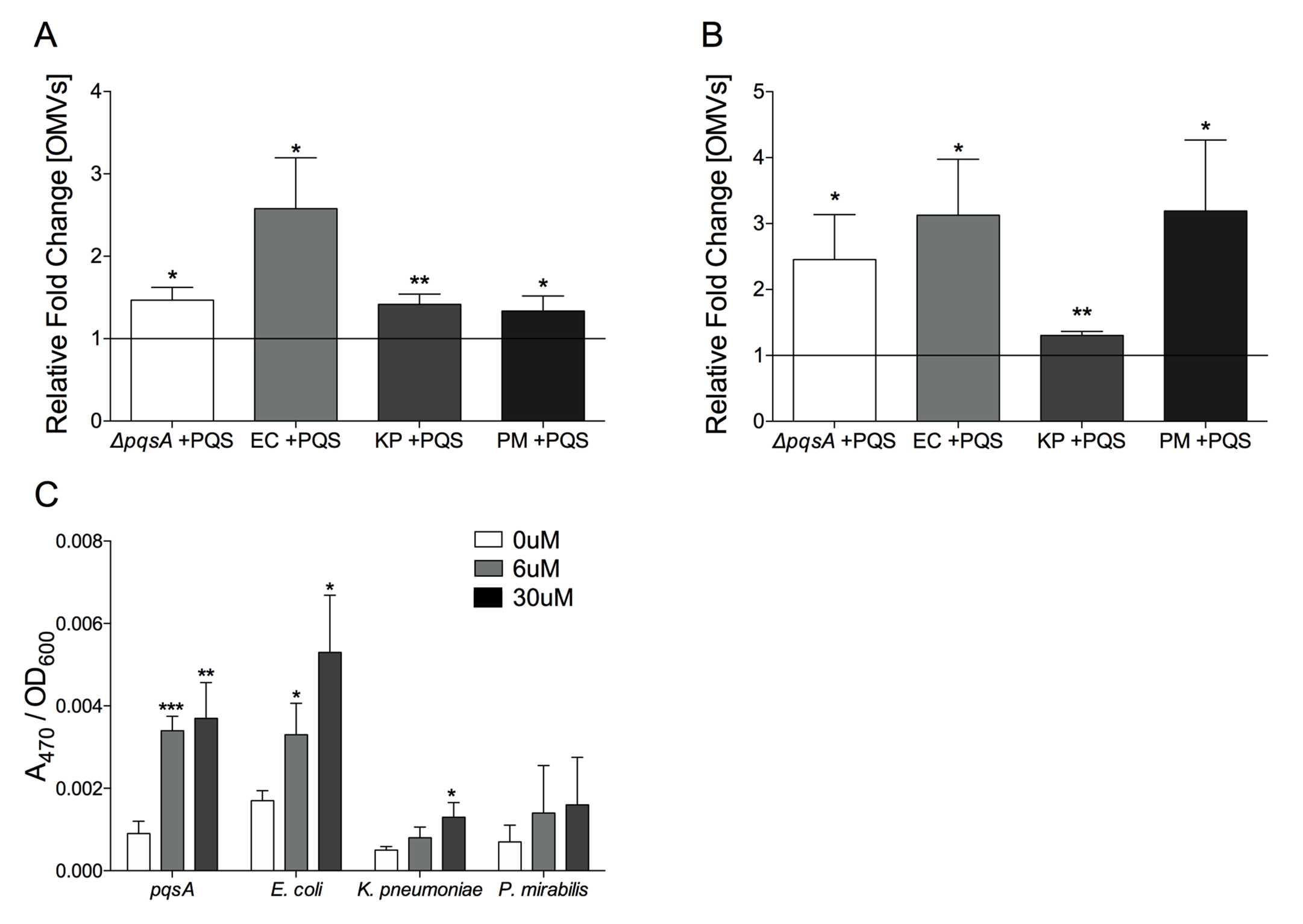
**

**Figure S3:** Fold increase in OMV production for all Gammaproteobacteria in the presence of exogenous PQS (relative to 0µM PQS for each corresponding strain). For comparison to data presented in figure 2d, fold change here was calculated using (a) the total of all particles of any size produced by recipient bacteria, or (b) the particles produced at only the most common (mode) 10 nm “bin” size. (c) Raw lipid assay data for all strains exposed to PQS in defined medium (MOPS). Exogenous [PQS] in “+PQS” = 6.7 ± 1.4 µM. Statistical significance was analyzed by one Tailed Student t-Test. (*** p < 0.001, ** p < 0.01, * p < 0.05, n > 3).
